# Supplementary material for: The kinetics of carbon pair formation in silicon prohibits reaching thermal equilibrium
Source: Nat Commun. 2023 Jan 23;14:361. doi: 10.1038/s41467-023-36090-2 (PMC9870972; doi:10.1038/s41467-023-36090-2)
Supplement: Supplementary file 1 — Supplementary Information [file 41467_2023_36090_MOESM1_ESM.pdf]

# Supplementary Information

# The kinetics of carbon pair formation in silicon prohibits reaching thermal equilibrium

Péter Deák,[1] Péter Udvarhelyi,[1,2] Gergő Thiering,[1] and Adam Gali[1,2]

[1] *Wigner Research Centre for Physics, P.O. Box 49, H-1525 Budapest, Hungary*

[2] *Budapest University of Technology and Economics, Műegyetem rkp. 3., 1111 Budapest, Hungary*

## SUPPLEMENTARY NOTE 1: DEFECT LEVEL STRUCTURES

The formation of the different configurations of the C-configuration of the dicarbon defect in silicon can be formulated as reactions of single atomic defects diffusing inside the cell. We consider the following reactions in the main text

$$\text{(Si-Si)}_{\text{Si}} + \text{C}_{\text{Si}} \rightarrow \text{(C-Si)}_{\text{Si}} \qquad -1.86\,\text{eV}, \tag{1}$$

$$\text{(C-Si)}_{\text{Si}} + \text{(C-Si)}_{\text{Si}} \rightarrow \text{C-configuration} + \text{(Si-Si)}_{\text{Si}} \qquad +0.39\,\text{eV}, \tag{2}$$

$$\text{(C-Si)}_{\text{Si}} + \text{C}_{\text{Si}} \rightarrow \text{A-configuration} \qquad -0.96\,\text{eV}, \tag{3}$$

$$\text{(C-Si)}_{\text{Si}} + \text{C}_{\text{Si}} \rightarrow \text{C-configuration} \qquad -1.47\,\text{eV}, \tag{4}$$

where we summarize here the binding energy of the reactions as obtained in the 512-atom simple cubic supercells. Negative(positive) values represent exothermic(endothermic) reactions, respectively. For the binding energy of the critical (3) and (4) reactions, we obtained −1.06 eV and −1.52 eV, respectively, that are within 0.1 eV with those obtained in the 512-atom supercell. We conclude that the 64-atom supercell yields sufficiently accurate results for the *relative* total energies of the critical defects which is the basis for the computationally demanding barrier energy calculations in the 64-atom supercell.

We plot the defect level structure (black lines) and their spin-polarized occupation (red arrows) inside the band structure of bulk silicon (cyan bands) for the three configurations of the dicarbon defect discussed in the main text (see Supplementary Figure 1) and the most important constituent defects in the above formation process (see Supplementary Figure 2). We show only the neutral ground state configurations. The optimized geometries of $\text{C}_{\text{Si}}$ and $\text{(Si-Si)}_{\text{Si}}$ defects are visualized in Supplementary Figure 3.

## SUPPLEMENTARY NOTE 2: HELMHOLTZ FREE ENERGY CALCULATION

For the accurate description of the formation of dicarbon defect in the A-configuration from the reaction of a substitutional carbon defect encountering a diffusing carbon split interstitial, we calculate the Helmholtz free energy of vibrations as a correction to the total energy of the defects. To this end, we calculate the vibrational spectrum of the defects in a 64-atom silicon supercell using PBE functional (see Supplementary Figure 4). In order to achieve consistent results, the minimum energy path was recalculated within PBE functional, and the appropriate vibration calculations were carried for the initial, final as well as the saddle point configurations at PBE level. The observed minimum energy path was similar to that obtained by HSE06 functional, thus we assume that the calculated vibrations at the given PBE geometries can provide a very good estimate for the *difference* of the vibriation entropy contributions. The calculated barrier energy by PBE is a bit smaller at 1.56 eV than the one by HSE06 (1.86 eV) which is typical due to the band gap error of PBE functional.

The Helmholtz free energy is calculated as

$$F_{\text{vib}} = U_{\text{vib}} - TS_{\text{vib}} = \sum_i \left[ \frac{1}{2}\hbar\omega_i + k_{\text{B}}T \ln\left(1 - e^{-\frac{\hbar\omega_i}{k_{\text{B}}T}}\right) \right], \tag{5}$$

where $\omega_i$ are the vibrational normal frequencies and $k_{\text{B}}$ is the Boltzmann constant. Our calculations at $T = 100$ °C result in correction energies of 0.012 eV and −0.132 eV for the energy barrier in the A-configuration defect formation and dissociation processes, respectively. So the activation energy for the dissociation is decreased from the original total energy difference of 1.86 eV to the vibrational free energy corrected value of 1.728 eV by assuming that the vibration entropy differences are well calculated at PBE level.

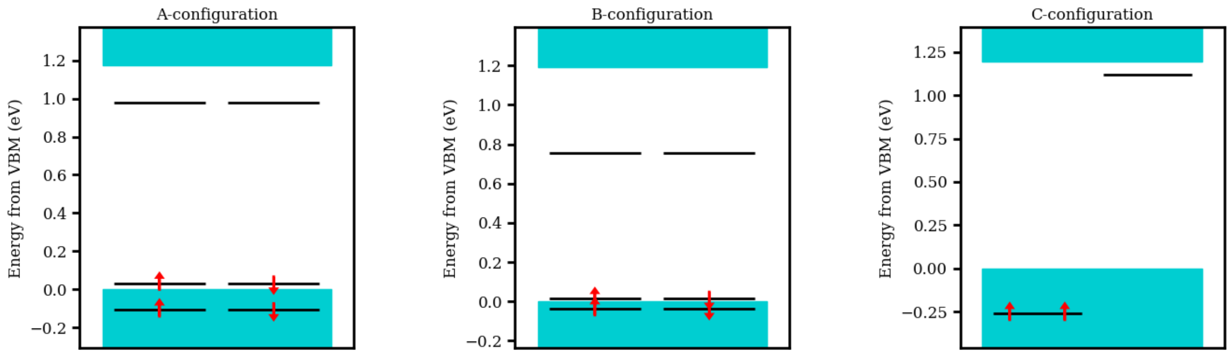


Supplementary Figure 1. HSE06 calculated Kohn-Sham level structure of the different configurations of the neutral dicarbon defect in 512-atom silicon supercell. See the main text for the optimized geometry of these dicarbon defects.

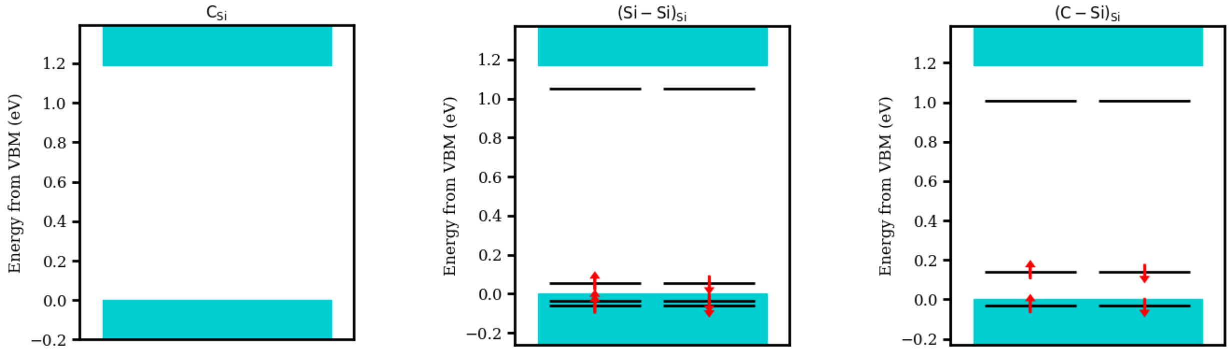


Supplementary Figure 2. HSE06 calculated Kohn-Sham level structure of the neutral carbon substitutional ($C_{Si}$), silicon interstitial [$Si_i$ or $(Si\text{-}Si)_{Si}$] and carbon split-interstitial [$(C\text{-}Si)_{Si}$] defects in 512-atom silicon supercell.

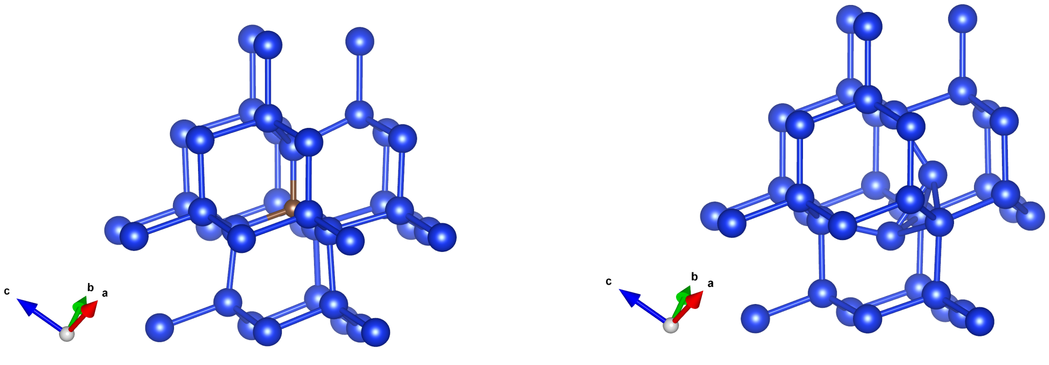


Supplementary Figure 3. Geometries off the carbon substitutional (left) and silicon split-interstitial defects in silicon.

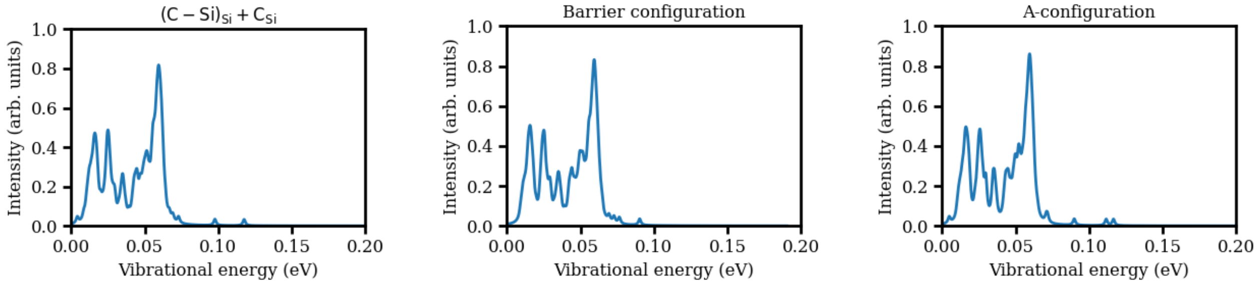


Supplementary Figure 4. PBE calculated vibrational spectrum for the initial, saddle point and final configurations in the reaction $(\mathrm{C}-\mathrm{Si})_{\mathrm{Si}}+\mathrm{C}_{\mathrm{Si}} \rightarrow$ A-configuration, in 64-atom silicon supercell.
